# Critical Insights: Hospital Admissions for Eating Disorders in Spanish Youth – A National Study

**DOI:** 10.1192/j.eurpsy.2025.1402

**Published:** 2025-08-26

**Authors:** A. Muñoz San Jose, P. Vázquez-Giraldo, T. López-Cuadrado

**Affiliations:** 1 Department of Psychiatry, Clinical Psychology, and Mental Health, La Paz University Hospital; 2 Department of Psychiatry, Autonomous University of Madrid (UAM); 3 Psychiatry and Mental Health Group, Neurosciences Research Area, Hospital La Paz Institute for Health Research (IdiPAZ), Madrid; 4 Centre of Biomedical Research in Mental Health (CIBERSAM: CB/07/09/0013), Institute of Health Carlos III, Spain; 5 Department of Chronic Diseases Epidemiology, National Centre for Epidemiology, Institute of Health Carlos III; 6 Department of Preventive Medicine and Public Health, Autonomous University of Madrid (UAM), Madrid, Spain

## Abstract

**Introduction:**

Eating disorders (EDs) are complex conditions with significant impacts on physical and mental health. This study investigates hospital admissions and resource utilization for EDs among pediatric patients in Spain over a seven-year period, emphasizing the effects of the COVID-19 pandemic.

**Objectives:**

To analyze the epidemiological trends, prevalence, and hospital resource use for pediatric ED admissions in Spain from 2016 to 2022.

**Methods:**

A retrospective analysis was conducted using data from Spain’s Minimum Basic Data Set of acute hospitals (CMBD-H) for patients aged 10–19 years with an ED diagnosis (ICD-10-CM code F50) between January 1, 2016, and December 31, 2022. Trends in hospitalization rates, types of EDs, comorbidities, and resource utilization were assessed.

**Results:**

The study identified 8,275 pediatric ED admissions, comprising 17% of all mental disorder admissions. **Table 1** summarizes the characteristics of hospital admissions by age group (10-14 years and 15-19 years). Hospitalization rates increased from 19.4 per 100,000 in 2016 to 32.7 in 2022, with a notable rise in the 10–14-year age group post-pandemic. Anorexia Nervosa (AN) was the predominant diagnosis (71.6%). Psychiatric comorbidities were present in 35.6% of cases. The average length of stay was 24 days, with annual direct medical costs totaling €3 million and a median cost per admission of €11,580. AN patients had longer hospital stays (median 28 days) compared to other disorders, while Bulimia Nervosa (BN) patients had shorter stays (median 14 days) but higher average costs (€12,300). **Table 2** details the average hospital length of stay and average cost of hospital admissions for EDs among adolescents in Spain.

**Image 1:**

**Figure:**
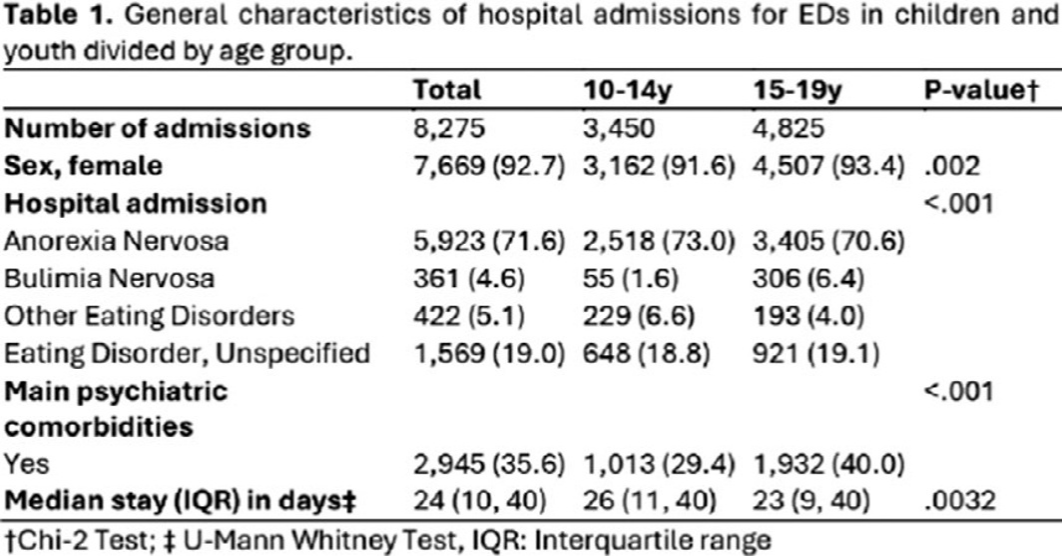

**Image 2:**

**Figure:**
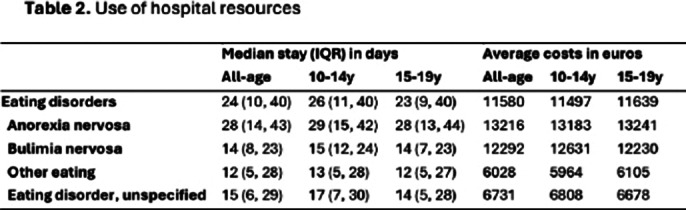

**Conclusions:**

There was a 70% increase in pediatric ED admissions in Spain from 2016 to 2022, with a significant post-pandemic rise in younger patients. This underscores the need for improved early detection and intervention strategies, as well as enhanced screening for comorbid mental health conditions to manage the growing hospital demand effectively.

**Disclosure of Interest:**

None Declared

